# Characterizing Herbal Medicine Use for Noncommunicable Diseases in Urban South Africa

**DOI:** 10.1155/2015/736074

**Published:** 2015-10-18

**Authors:** Gail D. Hughes, Oluwaseyi M. Aboyade, Roxanne Beauclair, Oluchi N. Mbamalu, Thandi R. Puoane

**Affiliations:** ^1^South African Herbal Science and Medicine Institute (SAHSMI), Faculty of Natural Sciences, University of the Western Cape, Private Bag X17, Bellville 7535, South Africa; ^2^South African Herbal Science and Medicine Institute, University of the Western Cape, Bellville 7535, South Africa; ^3^The South African Centre for Epidemiological Modelling and Analysis, Stellenbosch University, Stellenbosch 7602, South Africa; ^4^International Centre for Reproductive Health (ICRH), Ghent University, De Pintelaan 185 UZP114, 9000 Gent, Belgium; ^5^School of Pharmacy, University of the Western Cape, Bellville 7535, South Africa; ^6^School of Public Health, University of the Western Cape, Bellville 7535, South Africa

## Abstract

Economic challenges associated with noncommunicable diseases (NCDs) and the sociocultural outlook of many patients especially in Africa have increased dependence on traditional herbal medicines (THMs) for these diseases. A cross-sectional descriptive study designed to determine the prevalence of and reasons for THM use in the management of NCDs among South African adults was conducted in an urban, economically disadvantaged area of Cape Town, South Africa. In a cohort of 1030 participants recruited as part of the existing Prospective Urban and Rural Epidemiological (PURE) study, 456 individuals were identified. The overall prevalence of THM use was 27%, of which 61% was for NCDs. Participants used THM because of a family history (49%) and sociocultural beliefs (33%). Hypertensive medication was most commonly used concurrently with THM. Healthcare professionals need to be aware of the potential dualistic use of THM and conventional drugs by patients, as this could significantly influence health outcomes. Efforts should be made to educate patients on the potential for drug/herb interactions.

## 1. Introduction

The 2014 Global Status Report on noncommunicable diseases (NCDs) by the World Health Organization [1] indicates that these diseases currently cause more deaths than all other causes combined. These deaths are projected to increase from 38 million in 2012 to 52 million by 2030 [1]. This report also showed that four major NCDs (cardiovascular diseases, cancer, chronic respiratory diseases, and diabetes) are responsible for 82% of NCD deaths [1]. About 75% of all NCD deaths occur in low- and middle-income countries, usually in patients younger than 70 years old, and in the age range which constitutes the bulk of the work force [1–4]. This is a major health and development challenge for the 21st century [5–7].

In South Africa, the major NCDs are cardiovascular diseases, diabetes, cancers, chronic respiratory diseases, and mental illness [8]. The South African healthcare system, already under considerable pressure because of the high prevalence of tuberculosis and HIV/AIDS, has to contend with the burden of NCDs [9]. This burden affects the individual's quality of life and has resulted in increased healthcare expenses, not only financially, but also in terms of morbidity, at the individual and national levels [6, 10–12]. Recent updates indicate that South Africa is going through an epidemiological shift, with deaths occurring mainly as a result of NCDs [13].

Risk factors associated with the development of NCDs include heredity, unhealthy diets, a generally sedentary lifestyle and environment, and exposure to tobacco and alcoholic products. Such risk factors, caused by pressures of urbanization and modernization, have resulted in an increased burden to the struggling healthcare systems of developing countries [14]. Many of the affected citizens in these countries are unable to afford primary healthcare treatment for NCDs; hence, they depend, even if minimally, on alternative therapies such as traditional herbal medicines (THMs).

In certain parts of Africa, THMs still remain the most utilised form of healthcare because of their accessibility to the community [15]. Some physicians have reportedly recommended nonorthodox healing methods, such as traditional (herbal) healing to their patients, sometimes in cases where orthodox methods and treatment have not shown improvement [16, 17]. The various uses of many traditional herbal products have also been validated by* in vitro* studies, which may have informed such recommendations. The World Health Organization, in recognition of the role of THMs especially in resource-constrained settings, has called for preservation and acknowledgement of THM use in cases where such use has been scientifically validated [18]. The significance of this, in the face of the increasing global practice of medical pluralism (the adoption of more than one medical system), cannot be overlooked.

Surveys conducted in South Africa revealed that patients admit to using THMs for conditions such as diabetes, high blood pressure, sexually transmitted diseases, asthma, pain, HIV/AIDS, gynaecological and obstetric complaints, and childhood diseases [19–25]. In their study among Indians in Durban, South Africa, Singh et al. [26] reported that herbal/natural medicines were the most commonly used complementary and alternative medicines (CAM) to manage NCDs such as diabetes, arthritis, hypertension, and respiratory disorders, sometimes in conjunction with conventional medicines.

Despite the wide use of THMs, there is limited data on the prevalence of using these for the treatment of NCDs among people living in different regions of South Africa. This is certainly of importance to all stakeholders—patients, physicians, and government—as there exists potential for prescription drug-traditional medicine interaction, which may be beneficial or detrimental to patients. Therefore, this study characterized the use of THM in a selected urban area of Cape Town, South Africa, and examined potential predictors of use and the relationship between diagnosed NCDs and THM use.

## 2. Methods

### 2.1. Study Design

A cross-sectional descriptive study was conducted, and subjects were conveniently recruited from the South African arm of a larger prospective study, the Prospective Urban and Rural Epidemiological (PURE) study. For the PURE study, a global cohort has been developed to investigate the impact of social and environmental transition on health, involving over 150,000 adults initially aged between 35 and 75 years from communities in 17 low-, middle-, and high-income countries. A detailed description of the PURE study design has been published by Teo et al. [27].

### 2.2. Study Setting

For the THM aspect of the PURE study, South Africa, urban participants residing in Langa, a black community located in Cape Town in the Western Cape Province, were recruited. Langa, the oldest African township in Cape Town, has a high population of migrant black South Africans, who initially settled here because of lower living costs, proximity to the city, and available transport resources [28]. Most of the respondents were born in Langa. Like many other townships in Cape Town, it is one of the poorest areas as determined from the City Development Index and Human Development Index values which are below the provincial average [29, 30]. Formal houses exist along with informal settlements (the Joe Slovo informal settlement in Langa is one of the largest informal settlements in the country), unemployment is high, and the education level is generally below matric level [31].

### 2.3. Sampling for THM Study

Sampling for the PURE-THM study was conducted according to methods reported by Teo et al. [27]. The sampling frame for the current THM study made use of the 1030 participants who were recruited from Langa in the urban South Africa cohort of the PURE parent study. Participants' information captured throughout the PURE follow-up period was used to conveniently select 458 individuals who were originally enrolled in the PURE parent study and who had reported having at least one NCD. Those recruited were subsequently interviewed to determine the prevalence of THM use. Two of the 458 individuals were excluded from the analysis, because they did not answer the question, “Do you use THM?” Participants' names, contact details, and residential addresses were noted to facilitate the process of data collection. All individuals who agreed to participate provided written informed consent.

### 2.4. Data Collection

Appointments to collect data were first made with participants by means of telephone calls. Using the participants' residential addresses, trained interviewers visited the households/individuals on the day of the appointment to collect data on the epidemiology of THM use for NCDs. For the purpose of this study, data were collected between October 2013 and August 2014 using structured questionnaires which were administered through face-to-face interviews. The interviews were conducted by five trained data collectors in the preferred language of the respondent (English or isiXhosa). Participants residing in Langa, with origins mainly from the Eastern Cape Province of South Africa, speak isiXhosa and preferred to be interviewed and to respond in their mother tongue. This cohort is established, most of them having been born in Langa; however, participants regularly travel back to the Eastern Cape Province of origin to see family members. Data were collected about the respondents' demographic characteristics (age, sex, education, and employment status), clinical/medical history, traditional medicine use (duration of use, condition for use, dosage, and form), and migratory status. The quality of data collected was maintained through the use of standardized protocols and centralized training.

### 2.5. Data Analysis

Statistical analysis was performed using R statistical programming language, version 3.1.1 [32]. Initially, the frequency distributions and summary statistics for participant attributes and characteristics of THM use were computed. Participants were classified as THM users if they answered “Yes” to the question “Do you use traditional herbal medicine?”

Next, a bivariate analysis of eight different self-reported conditions and THM use was conducted. The conditions were hypertension, diabetes, rheumatoid arthritis, cardiovascular disease, heart disease, depression, hypercholesterolemia, and asthma. Some conditions were rare and thus had low frequencies; therefore, Fisher's exact tests were employed to determine statistical significance. The proportion of THM users who concurrently used specific classes of conventional medicines, antihypertensives, diuretics, medicines for pain, anti-inflammatory agents, and antidiabetic and cholesterol-reducing agents, were also graphically presented.

Finally, 18 variables were explored as potential predictors of THM use:* Gender* (male/female),* Marital status* (never married/married or cohabiting/divorced, widowed, or separated),* Education* (none or primary/secondary/tertiary or other),* Employed* (Yes/No),* Income per month* (R0–R1999 [0–163 USD]/R2000–R5000 [163–408 USD]/>R5000 [>408 USD]),* Religion* (Christian/Other),* Medical insurance* (Yes/No),* Age* (numeric, continuous),* Number of people in household* (numeric, discrete),* Health compared to last year* (same as last year/better than 1 year ago/worse than 1 year ago),* Have current health condition* (No/Yes),* Uses conventional medication* (No/Yes),* Sees a family doctor* (No/Yes),* Sees a specialist doctor* (No/Yes),* Sees a hospital doctor* (No/Yes),* Sees a traditional healer* (No/Yes),* Has a noncommunicable disease* (No/Yes), and* Migrant* (Yes/No).* Has a noncommunicable disease* and* Migrant* were constructed variables. Participants were classified as having a noncommunicable disease if they reported having any of the following conditions: hypertension, diabetes, stroke, rheumatoid arthritis, cardiovascular disease, heart disease, depression, hypercholesterolemia, and asthma. For the* Migrant* variable, participants were coded as “Yes,” if they reported living in another province different from their province of origin or birth (i.e., migrating between provinces). These 18 variables were considered because of the study team's* a priori* hypotheses that these may influence a person's use of THM. Logistic regression was used to calculate crude odds ratios (OR) and 95% confidence intervals (95% CI). However, none of these models were adjusted for other variables as the research focus was to determine marginal associations with THM use.

## 3. Results

A total of 456 participants were included in the analyses. Characteristics of these participants are presented in Table 1. The median age of participants was 56 years, and over one-quarter of the participants (27.2%, *n* = 124) reported using THM. Most participants were female (78.0%, *n* = 355), had at least a secondary education (63.0%, *n* = 283), and were identified with the Christian religion (95.8%, *n* = 431). About half of the participants had never been married (49.8%, *n* = 223). Only 15.6% and 4.1% of the study participants were employed and had medical insurance, respectively.

Table 2 presents characteristics of THM use among the 124 self-ascribed users in our study sample. The median age at first use of THM was 35 years and over half of these users had used THM for several years or were unsure of how long they had been using these (26.9% and 28.6%, resp.). The THMs were obtained from the markets (39.5%) and traditional health practitioners (THPs) (26.6%) or were personally harvested by the participating user (21.0%).

The preferred mode of THM preparation was as a tea for oral consumption (83.9%), as opposed to an extract (10.2%), powder (9.3%), decoction (6.8%), or tablet (2.5%). More than half of the participants (57.8%) reported that relatives influenced them to use THM, and the most common reasons for using THM were ascribed to family history (48.8%) and cultural beliefs (33.3%). The percentage of participants who believe in the efficacy of THM was 64.7, with such efficacy rated by 47.5% of THM users as equal to or more than that of conventional medicines. The practice of medical pluralism is evident, with 37.1% of THM users admitting to concurrent use of THM with their conventional medicines. Also, 61.3% of people who use THM self-reported diagnosis of an NCD.

**Figure 1:**
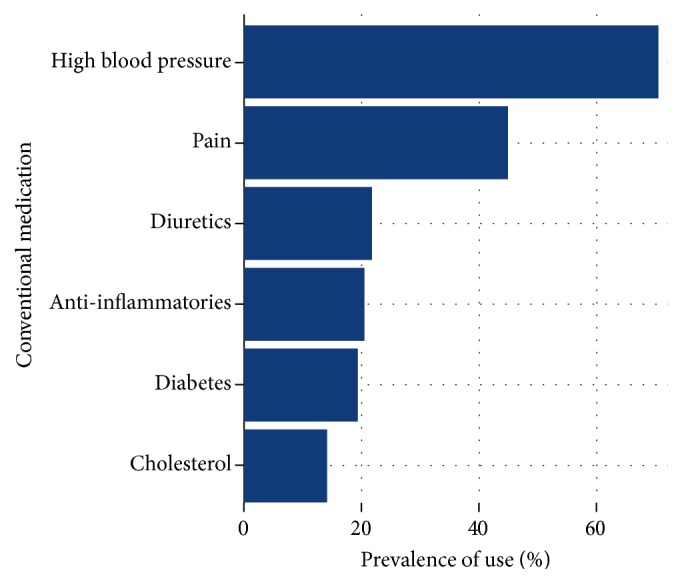
Proportion of THM users who are also using different types of conventional medicines.

Figure 1 depicts the proportion of THM users who also used different types of conventional medicines. Among the THM users, the highest prevalence of medical pluralism (specifically, concurrent THM and conventional medicine use) was noted among participants who used conventional medicines for high blood pressure and pain.

The associations between different reported conditions and THM use can be seen in Table 3. The only statistically significant relationship observed is that between having rheumatoid arthritis and using THM (*p* < 0.05). Although not statistically significant, a greater proportion of THM users were hypertensive (53.2%) compared to nonusers of THM who were hypertensive (47.3%).

Finally, Table 4 presents the predictors of THM use as determined by logistic regression analysis.

For every year when participants' age increases, there appears to be 1% increased odds of using THM (95% CI: 0.99–1.03). Table 1 reflects the median age for THM users as 57, compared to 56 among nonusers. Participants with a secondary education are 44% less likely to use THM than people with no education or primary education (95% CI: 0.36–0.88). Corroborating this, Table 1 shows that two-thirds of nonusers have a secondary education versus only 54.5% of THM users. Those who said their health was better at the time of the survey than the previous year were more likely to have used THM than those who claimed their health was the same as in the year prior to the survey (OR 1.72, 95% CI: 1.04–2.91). Participants who reported a current health condition had approximately two times the odds of using THM compared to those without (95% CI: 1.28–3.34). Consultations with a family doctor (OR 2.26, 95% CI: 1.48–3.46), specialist physician (OR 2.38, 95% CI: 1.40–4.00), or a traditional healer (OR 8.66, 95% CI: 3.50–24.52) were predictors of using THM. Participants who were currently living in their province of birth or origin had 41% reduced odds of using THM (95% CI: 0.39–0.90) compared to migrants. Of those using THM, more than half (53.2) were classified as migrants (Table 1).

## 4. Discussion

The aim of this study was to understand the prevalence of THM use for NCDs in an urban township in the Western Cape Province of South Africa. The township has been in existence for over 100 years and most of the respondents were born there. The prevalence of THM use observed in this study falls within the range, 6.1%–38.5%, documented in the systematic review of THM/CAM use in South Africa conducted by Peltzer [33]. This is however lower than what has been reported in other South African studies [20, 26] and in studies conducted in different settings and countries within Africa such as in Nigeria [34], Ghana [35], and Uganda [36], and further afield in Korea [37], Turkey [38], Finland [39], and Australia [40]. The observed difference in prevalence of THM use might be as a result of variation in sample characteristics, study setting, and population.

Patterns of THM acquisition recorded in this study bear some similarity to another study conducted in Ghana [41]. For instance, in this study, the major sources of THM for many of the users were the market (39.5%), the traditional health practitioner (26.6%), and personal harvest (21.0%) while for the Ghanaian study, the major sources of CAM products, most of which were herbal medicinal products, were personal harvest (37/3%), the market (21.6%), and the pharmacy (11.8%). The high percentage of participants in this study who obtain THM from the market and their personal harvest implies that many of the participants self-prescribed and could identify these medicines (for purchases from the market and personal harvest). The percentage of participants who obtain their THM from the pharmacy with respect to this study (13.7%) and the Ghanaian study (11.8%) perhaps implies increasing confidence in such products, seeing that they can be obtained from the same place as conventional drugs. This raises concern regarding the quality and potential interactions of these products and the need for the pharmacist to educate patients and customers regarding this. Since less than half (26.6%) of the THM users in this study obtained their THM from THPs, it can be assumed that the practice of self-diagnosis and self-medication with traditional medicine is widespread in urban areas of developing countries. Self-medication with THM has also been observed among all age groups and social categories of people [42, 43].

This study observed that drinking the THM as a tea was the most common mode of THM consumption, which raises questions regarding the quality and stability profiles of the prepared remedy on storage. The use of THM is largely influenced by family and cultural reasons. Interestingly, while literature sources have reported that many people utilized THMs because they were more affordable and accessible than conventional treatment methods [44–47], less than 30% of the present study participants utilized THMs for these two reasons, in spite of the high percentage of unemployment and low-income level of the participants. In the urban community where this study was set, THMs are used mainly because of their sociocultural acceptance, largely influenced by family and the respondent's satisfaction with THM. This is also reflected in the percentage of THM users who believed that THM not only was effective but also had an efficacy which was equal to or greater than that of conventional medicines (Table 2). The results of this study imply that THM use may have more to do with the participant's health beliefs and family history of use compared to sociodemographic attributes such as income and employment.

More than 90% of respondents reported being Christians, and this applied to both users and nonusers of THM alike. This perhaps shows that respondents still adhere to their sociocultural heritage and do not see it as opposing to their spiritual views. Indeed, spirituality has been documented as a strong predictor of traditional or complementary and alternative medicine use [48]. Studies in countries such as Sierra Leone, India, and Malaysia also bear credence to this, with many CAM and allopathic practitioners believing that there was an increase in spiritual focus among patients when they are ill compared to when they are healthier. Individuals reportedly receive religious support during illness, and many allopathic and traditional or CAM practitioners believe this improves health outcomes [49–51].

Although our study indicated no statistically significant difference between diagnosed conditions and THM use, with the exception of rheumatoid arthritis, a high prevalence of THM use was observed among patients who had hypertension. Previous studies have also documented evidence of common use of CAM such as THM among hypertensive patients [41, 52]. Rheumatoid arthritis is perhaps viewed, and perhaps wrongly too, as one of the least threatening NCDs without an urgent need for treatment/management as required for NCDs like hypertension and diabetes. Many patients do not receive timely and appropriate therapy for the management of this condition. This has been attributed to difficulty in making a diagnosis among physicians who are not specialized in rheumatology, unlike the diagnosis of other NCDs like hypertension and diabetes which can easily be made and managed even by nonspecialist physicians [53]. This perhaps leads to participants' exploration of CAM and significantly increases the likelihood of THM use by patients with rheumatoid arthritis, compared to those with other NCDs such as hypertension and diabetes, which can be easily diagnosed. Patients with rheumatoid arthritis were also more likely than patients with hypertension or diabetes to underutilize their prescription medications as part of cost-cutting measures [54], which may predispose them to use of nonprescription alternative medicines such as THM.

Moreover, participants did not use THMs to treat or manage a condition. This lends support to findings from previous studies where individuals are documented to use CAM treatment measures, such as THM to improve immunity and promote general well-being [22, 55]. While some participants indicated the frequent use of THM, majority utilized THM rarely. This may perhaps be explained by the premise that THMs are not used exclusively, but in combination with and as a supplement to conventional medicines, a view which is supported by Cook [56] and Singh et al. [26].

Medical pluralism was evident among hypertensive patients. Given that diuretics are a major class of drugs utilized to control hypertension, the prevalence of use among hypertensive patients may be higher than presented in the figure. The same interpretation may also be made for the prevalence of medical pluralism among patients who use conventional medicines for pain and inflammation. Previous studies have also hinted that patients with NCDs may use THM for the treatment of their condition and its related side effects as well as for other unrelated self-limiting ailments [22, 52, 57]. The use of complementary treatment methods such as THMs is also quite common among individuals with NCDs in other parts of the world [58–60].

Unlike our previous study which found age and marital and employment status as predictors of THM use [22], the same relationship could not be established in the present study. This could be as a result of differences in the populations assessed as well as differences in the study locations. In the study population, participants with a secondary education are less likely to use THM than those with primary or postsecondary education. A similar study in Ghana also reported that respondents with a secondary education utilized THM less than those with basic or no education but less than those with a tertiary education [35]. Although people with poor health are generally more likely to seek alternative treatment methods such as THM [61–64], our study showed that participants who reported a better health status than the previous year were more likely to use THM. Participants who see a family doctor or a specialist doctor also have a greater likelihood of THM use than those who see hospital doctors. Possibly, these participants might have a patient-physician relationship which increases such odds, unlike participants who see a hospital doctor. Could the pressures that hospital doctors work under in public settings be an obstacle to the development of such relationship? No evidence was however found in the literature to support these views. A professional diagnosis possibly empowers these patients to know without question what their health problems are. This may enable them to access and assess THM practices for NCDs as opposed to patients who see a doctor, who may not be a specialist, at the local community clinic or hospital.

Postapartheid South Africa is undergoing urban migration. Although urban areas within the country are known to have better health infrastructures than the rural areas, exceptions to this fact are the urban areas with high concentration of slums and squatter settlements [65]. Migrant populations in this study were more likely to use THM compared to the nonmigrants. This is consistent with many other studies which document a higher prevalence of CAM measures such as THM among migrant and immigrant populations [42, 66–70].

In consultations with patients, physicians generally do not make enquiries regarding their patients' use of CAM, such as THM [40, 41]. There is a need to improve communication flow between physician and patient, especially patients with NCDs. This would make the physician aware of THM prevalence, as well as understand patients' health-seeking patterns. Data presented identifies four major factors—basic or no education, individual view of health status, relationship with family/specialist doctor, and migratory history—that may predict a patient's likelihood of using THM. These predictors may serve as indicators (of THM use) to physicians during consultations with patients, thus enabling them to introduce appropriate education and intervention programmes to assist patients in making informed decisions regarding their use of THM.

## 5. Study Strengths and Limitations

This study represents a timely investigation of THM use in black African participants with NCDs living in a South African urban setting. A strong case is made with its multidisciplinary approach which focuses on THM use and its public health implications. Being a cross-sectional study, it has indicated associations between some of the assessed predictors and THM use. This is the first analysis of THM prevalence and predictors of use among patients in this community, the results of which may be used as baseline data for future studies.

We acknowledge that this study had some limitations. The cross-sectional nature of the study means that causality may not be developed between the exposure and outcome. In addition, while face-to-face interviews along with trained data capturers and the employment of a centralized training system may serve to improve quality of data collected, it may also suffer from social desirability and self-report bias on the part of the participant. Therefore, the fact that the validity of our findings may be subjective, depending as it was on the participants' ability to recall as well as present accurate information with respect to their THM use, cannot be dismissed. In addition, the study population was from a township in an urban area and so results many not be generalized to other townships or rural areas.

## 6. Conclusions

The results of this study highlight the prevalence of and patterns of use of THMs in an urban South African community. Generally, THM use is unsupervised via the South African health system. Information on THM is largely sourced from family, and the practice of self-medication is common. Of concern from the results of this study is the prevalence of THM coutilization with conventional medicines. Records of potential drug interactions and contraindications for the use of THMs are not yet available. Nevertheless, it is of important public health interest to make health workers aware of patients' THM use and the potential for prescription drug-THM interaction and its clinical significance. Such awareness will enable them to offer appropriate advice regarding the use of these products.

Given the diverse multicultural and multiethnic orientation of the South African population, it would be of great public and health interest to conduct similar studies among other cultural and ethnic groups.

## Figures and Tables

**Table 1:** Participant characteristics.

	Overall *n* = 456	THM use *n* = 124	No THM use *n* = 332
THM use *n* (%)			
Yes	124 (27.2)		
No	332 (72.8)		
Gender *n* (%)			
Male	100 (22.0)	25 (20.3)	75 (22.6)
Female	355 (78.0)	98 (79.7)	257 (77.4)
Marital status *n* (%)			
Never married	223 (49.8)	50 (42.0)	173 (52.6)
Married or cohabiting	127 (28.3)	38 (31.9)	89 (27.1)
Divorced, widowed, or separated	98 (21.9)	31 (26.1)	67 (20.4)
Education *n* (%)			
None or primary	129 (28.7)	46 (37.4)	83 (25.5)
Secondary	283 (63.0)	67 (54.5)	216 (66.3)
Tertiary or other	37 (8.2)	10 (8.1)	27 (8.3)
Employed *n* (%)			
Yes	68 (15.6)	23 (19.7)	45 (14.2)
No	367 (84.4)	94 (80.3)	273 (85.8)
Income *n* (%)			
R0–R1999	369 (81.6)	93 (75.0)	276 (84.1)
R2000–R5000	73 (16.2)	26 (21.0)	47 (14.3)
R5000+	10 (2.2)	5 (4.0)	5 (1.5)
Religion *n* (%)			
Other	19 (4.2)	7 (5.6)	12 (3.70)
Christian	431 (95.8)	117 (94.4)	314 (96.3)
Medical insurance *n* (%)			
Yes	18 (4.1)	8 (6.7)	10 (3.1)
No	426 (95.9)	111 (93.3)	315 (96.9)
Smoking status *n* (%)			
Never smoked	275 (62.2)	78 (66.7)	197 (60.6)
Past smoker	32 (7.2)	10 (8.5)	22 (6.8)
Current smoker	124 (28.1)	28 (23.9)	96 (29.5)
Casual smoker	11 (2.5)	1 (0.9)	10 (3.1)
Alcohol use *n* (%)			
Never drank	225 (50.6)	66 (55.9)	159 (48.6)
Past drinker	40 (9.0)	13 (11.0)	27 (8.3)
Current drinker	127 (28.5)	24 (20.3)	103 (31.5)
Casual drinker	53 (11.9)	15 (12.7)	38 (11.6)
General health *n* (%)			
Excellent	35 (7.8)	10 (8.4)	25 (7.6)
Very good	71 (15.9)	16 (13.4)	55 (16.8)
Good	176 (39.5)	55 (46.2)	121 (37.0)
Fair	106 (23.8)	23 (19.3)	83 (25.4)
Poor	58 (13.0)	15 (12.6)	43 (13.1)
Migrant *n* (%)			
No	170 (37.6)	58 (46.8)	112 (34.1)
Yes	282 (62.4)	66 (53.2)	216 (65.9)
Age med (IQR)	56 (47–64)	57 (49–64)	56 (46–64)
Number of people living in household med (IQR)	5 (3–6)	5 (3.8–7)	5 (3–6)
Number of people earning an income in household med (IQR)	1 (1-2)	1 (1-2)	1 (1-2)

**Table 2:** Characteristics of traditional herbal medicines (THMs) use.

Age when participant first used THM med (IQR)	35 (20–54)
Obtains THM at market *n* (%)	
Yes	49 (39.5)
No	75 (60.5)
Obtains THM at traditional practitioner *n* (%)	
Yes	33 (26.6)
No	91 (73.4)
Obtains THM from personal harvest *n* (%)	
Yes	26 (21.0)
No	98 (79.0)
Obtains THM from the pharmacist *n* (%)	
Yes	17 (13.7)
No	107 (86.3)
Obtains THM over the counter *n* (%)	
Yes	5 (4.0)
No	119 (96.0)
Participant takes THM as a tea *n* (%)	
Yes	99 (83.9)
No	19 (16.1)
Participant takes THM as a powder *n* (%)	
Yes	11 (9.3)
No	107 (90.7)
Participant takes THM as an extract *n* (%)	
Yes	12 (10.2)
No	106 (89.8)
Participant takes THM as a tablet	
Yes	3 (2.5)
No	115 (97.5)
Participant takes THM as a decoction	
Yes	8 (6.8)
No	110 (93.2)
Who or what influences participant to use THM? *n* (%)	
Friends/colleagues	21 (18.1)
Partner	7 (6.0)
Family/relatives	67 (57.8)
Self	7 (6.0)
Traditional practitioner	8 (6.9)
Advertisement	3 (2.6)
Healthcare providers	2 (1.7)
Other	1 (0.9)
Family history is the reason for THM use *n* (%)	
Yes	60 (48.8)
No	63 (51.2)
Cultural beliefs are the reason for THM use *n* (%)	
Yes	41 (33.3)
No	82 (66.7)
Low cost is the reason for THM use *n* (%)	
Yes	20 (16.3)
No	103 (83.7)
Accessibility is the reason for THM use *n* (%)	
Yes	15 (12.2)
No	108 (87.8)
A positive recommendation is the reason for THM use *n* (%)	
Yes	28 (22.8)
No	95 (77.2)
Treating a condition is the reason for THM use *n* (%)	
Yes	40 (32.5)
No	83 (67.5)
Managing a condition is the reason for THM use *n* (%)	
Yes	16 (13.0)
No	107 (87.0)
How often is THM used by the participant? *n* (%)	
Never	2 (1.6)
Rarely	33 (26.8)
Sometimes	46 (37.4)
Often	28 (22.8)
Always	14 (11.4)
Participant uses THM in combination with conventional medicine? *n* (%)	
Yes	46 (37.1)
No	78 (62.9)
Participant thinks THM is effective? *n* (%)	
Yes	75 (64.7)
No	20 (17.2)
Sometimes	21 (18.1)
Participant thinks THM is better than CM? *n* (%)	
Less efficacy	38 (31.1)
Equal efficacy	32 (26.2)
More efficacy	26 (21.3)
Unknown	26 (21.3)
How much is the family willing to pay for THM per year?	
<R100	23 (19.5)
R100–R250	14 (11.9)
R251–R500	7 (5.9)
R501–R750	4 (3.4)
R751–R1000	2 (1.7)
>R1000	6 (5.1)
Nothing	62 (52.5)

**Table 3:** Reported diagnosed conditions and traditional herbal medicines (THMs) use.

Variable	THM use		*p* value
	Yes *n* (%)	No *n* (%)	
High blood pressure/hypertension			
Yes	66 (53.2)	157 (47.3)	0.29
No	58 (46.8)	175 (52.7)	
Diabetes			
Yes	20 (16.1)	62 (18.7)	0.59
No	104 (83.9)	270 (81.3)	
Rheumatoid arthritis			
Yes	21 (16.9)	32 (9.6)	<0.05
No	103 (83.1)	300 (90.4)	
Cardiovascular disease			
Yes	2 (1.6)	4 (1.2)	0.67
No	122 (98.4)	328 (98.8)	
Heart diseases			
Yes	3 (2.4)	6 (1.8)	0.71
No	1221 (97.6)	326 (98.2)	
Depression			
Yes	4 (3.2)	14 (4.2)	0.80
No	120 (96.8)	318 (95.8)	
Hypercholesterolemia			
Yes	2 (1.6)	10 (3.0)	0.53
No	122 (98.4)	322 (97.0)	
Asthma			
Yes	4 (3.2)	13 (3.9)	1.00
No	120 (96.8)	319 (96.1)	

**Table 4:** Predictors of traditional herbal medicines use.

	Crude OR (95% CI)
Age	1.01 (0.99–1.03)
Gender	
Male	1.00
Female	1.14 (0.70–1.93)
Marital status	
Never married	1.00
Married or cohabiting	1.48 (0.90–2.42)
Divorced, widowed, or separated	1.60 (0.94–2.71)
Education	
None or primary	1.00
Secondary	0.56 (0.36–0.88)
Tertiary or other	0.67 (0.29–1.47)
Employed	
No	1.00
Yes	1.48 (0.84–2.56)
Income per month	
R0–R1999	1.00
R2000–R5000	1.64 (0.95–2.78)
>R5000	2.97 (0.81–10.89)
Religion	
Other	1.00
Christian	0.64 (0.25–1.75)
Medical insurance	
No	1.00
Yes	2.27 (0.84–5.90)
Number of people in household	1.00 (0.93–1.07)
Health compared to last year	
Same as last year	1.00
Better than 1 year ago	1.72 (1.04–2.91)
Worse than 1 year ago	1.60 (0.80–3.18)
Have current health condition	
No	1.00
Yes	2.04 (1.28–3.34)
Uses conventional medication	
No	1.00
Yes	1.17 (0.76–1.81)
Sees a family doctor	
No	1.00
Yes	2.26 (1.48–3.46)
Sees a hospital doctor	
No	1.00
Yes	0.67 (0.38–1.19)
Sees a specialist doctor	
No	1.00
Yes	2.38 (1.40–4.00)
Sees a traditional healer	
No	1.00
Yes	8.66 (3.50–24.52)
Has a noncommunicable disease	
No	1.00
Yes	1.35 (0.89–2.07)
Migrant	
Yes	1.00
No	0.59 (0.39–0.90)
